# Characterization and overproduction of cell-associated cholesterol oxidase ChoD from *Streptomyces lavendulae* YAKB-15

**DOI:** 10.1038/s41598-019-48132-1

**Published:** 2019-08-14

**Authors:** Keith Yamada, Arina Koroleva, Mitchell Laughlin, Niko Oksanen, Amir Akhgari, Vera Safronova, Elena Yakovleva, Vera Kolodyaznaya, Tatiana Buldakova, Mikko Metsä-Ketelä

**Affiliations:** 1 University of Turku, Department of Biochemistry, Turku, Finland; 2 Saint Petersburg State Chemical Pharmaceutical University, Department of Biotechnology, Saint Petersburg, Russia; 3 All-Russian Research Institute for Agricultural Microbiology, Saint Petersburg, Russia

**Keywords:** Biocatalysis, DNA sequencing, Metabolic engineering

## Abstract

Cholesterol oxidases are important enzymes with a wide range of applications from basic research to industry. In this study, we have discovered and described the first cell-associated cholesterol oxidase, ChoD, from *Streptomyces lavendulae* YAKB-15. This strain is a naturally high producer of ChoD, but only produces ChoD in a complex medium containing whole yeast cells. For characterization of ChoD, we acquired a draft genome sequence of *S*. *lavendulae* YAKB-15 and identified a gene product containing a flavin adenine dinucleotide binding motif, which could be responsible for the ChoD activity. The enzymatic activity was confirmed *in vitro* with histidine tagged ChoD produced in *Escherichia coli* TOP10, which lead to the determination of basic kinetic parameters with *K*_m_ 15.9 µM and *k*_cat_ 10.4/s. The optimum temperature and pH was 65 °C and 5, respectively. In order to increase the efficiency of production, we then expressed the cholesterol oxidase, *choD*, gene heterologously in *Streptomyces lividans* TK24 and *Streptomyces albus* J1074 using two different expression systems. In *S*. *albus* J1074, the ChoD activity was comparable to the wild type *S*. *lavendulae* YAKB-15, but importantly allowed production of ChoD without the presence of yeast cells.

## Introduction

**Figure 1:**
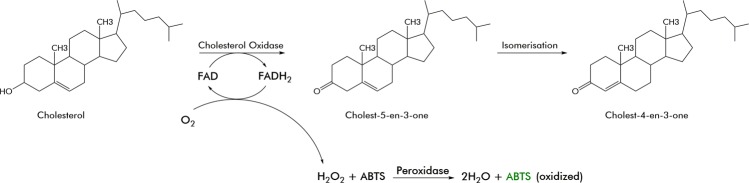
Cholesterol oxidase chemical reaction and activity assay reaction for the oxidation of ABTS.

Cholesterol oxidases (EC 1.1.3.6, 3β-hydroxysterol oxidase) are FAD-dependent (flavin adenine dinucleotide) enzymes, which oxidizes cholesterol to form cholest-4-en-3-one (cholestenone) and H_2_O_2_ (Fig. 1). Cholesterol oxidases have a broad range of applications including determination of food and serum cholesterol levels^1^, bioconversion of non-steroidal compounds^2^, allylic alcohols and sterols^2^, insecticidal activity^3,4^ and as a signal for the production of antifungal antibiotics^5^. Furthermore, cholesterol oxidases have been implicated in the manifestation of HIV, Alzheimer’s disease and tuberculosis^6^ and are needed for the biotransformation of cholesterol to cholestenone, which is an important precursor for the synthesis of hormones and steroidal drug intermediates^7^. More recently, cholesterol oxidases from *Borodetella* sp. have also been shown to promote cell apoptosis in lung adenocarcinoma^8^ and breast cancer^9^. Interestingly, enzymes extracted from *Streptomyces* sp. are typically preferred for industrial production as they are more stable than the ones isolated from *Nocardia* or *Pseudomonas*^10^.

Cholesterol oxidases in the context of basic biological function are utilized by various microorganisms to assimilate cholesterol as a carbon and energy source^11^. Two distinct classes of cholesterol oxidases have been found in microorganisms^1^. In many bacteria, including *Streptomyces* sp., cholesterol oxidases are intracellularly produced and secreted into the culture broth while in some others, e.g. *Nocardia* species, the enzyme is produced intrinsically membrane bound^1,12^. The intracellular proteins typically employ twin arginine transport (TAT) systems to export FAD-bound, fully folded proteins outside of cells, but the molecular details determining whether the proteins are bound to the extracellular matrix or secreted to the medium remain unresolved^13–15^. The strain *S*. *lavendulae* YAKB-15 has been noted to harbour significant cholesterol oxidase, ChoD, activity^16^, but appears to produce the enzyme only in a culture medium that contain whole yeast cells^17^. Originally this strain was cultivated with protein-vitamin concentrate as an organic nitrogen source, however, since it became unavailable other sources were tested (i.e. yeast extract, corn steep liquor, soy flour, peptone, casein hydrolysate, urea, beer yeast and baker’s yeast) and the use of baker’s yeast yielded the highest activity^16^. Interestingly, in contrast to several other strains of *Streptomyces*^4,5,9,18,19^, the activity is found associated with the fraction containing intact cells and only minor amounts can be detected from the supernatant.

In this study, we assembled a draft genome sequence of *S*. *lavendulae* YAKB-15 and identified a cholesterol oxidase, *choD*, gene in a putative operon (cho) containing two regulatory genes. Production of recombinant ChoD in *Escherichia coli* enabled the determination of kinetic parameters of the enzyme, whereas the overexpression in *S*. *albus* J1074 using a pSET-152-based vector^20^ led to comparative yields of ChoD production in comparison to *S*. *lavendulae* YAKB-15. Importantly, this allowed production of the ChoD in a simple medium without the presence of whole yeast cells.

## Results and Discussion

### Discovery of choD cholesterol oxidase from *S. lavendulae* YAKB-15

*Streptomyces lavendulae* YAKB-15 was found to produce ChoD under highly specific fermentation conditions^15,16^. The strain requires the presence of whole yeast cells, either live or autoclaved. No ChoD activity could be observed when yeast extract was used as a substitute in the production medium. In addition, the production appears to be strictly regulated in a temporal manner, with highest activity observed after 40 hours followed by rapid decline. Since these factors severely limit industrial strain development, we proceeded to obtain the draft genome sequence of *S*. *lavendulae* YAKB-15 and identify the gene responsible for ChoD production.

The sequencing data was acquired using MiSeq technology and resulted in 15,984,844 reads that were normalized, error corrected and trimmed down to 7,196,183 reads, which were then *de novo* assembled into 98 contigs. ABACAS ordered and aligned the contigs into 73 scaffolds with an N50 of 447,215 bp. The final genome assembly is 7.8 Mbp with a GC content of 72.2% and median coverage of 199x. The BUSCO analysis searched for 40 single-copy orthologs and found 36 (90%) were complete. Out of the 36 complete BUSCOs, 4 were found multiple times throughout the assembly and none were missing.

**Figure 2:**
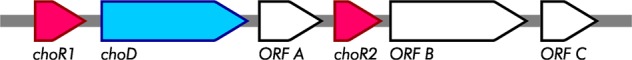
Putative cholesterol oxidase-containing operon (cho). The cholesterol oxidase (*choD*) gene is shown in blue. The two putative regulatory genes are shown in red, the remaining putative genes are shown in white.

BLAST analysis was used to identify putative cholesterol oxidases and led to the discovery of a gene denoted *choD* that showed 82% identity to a cholesterol oxidase gene from *Streptomyces* sp. SA-COO (UniProt P12676^21^). The high sequence identity suggests that ChoD belongs to the glucose-methanol-choline oxidoreductase family^22^ and possesses the classical Rossman fold for dinucleotide binding, which is found in many flavin-dependent oxidases^23^. Further analysis of the gene product showed that all of the important catalytic residues were conserved (e.g. E389 and H484) and the N-terminal region contained a twin-arginine transport (TAT) signal^21^, which displayed 25/42 (60%) identity to P12676. The *choD* gene resides in a putative operon structure with six genes (Fig. 2). Two of which are regulatory genes of the LuxR^24^ and PadR^25^ families (Table 1). Related proteins in these families have been found to control many aspects of secondary metabolism in *Streptomyces*, including antibiotic production and resistance^24–27^, and therefore could be responsible for the transient transcription of *choD*. In addition, the cho operon contained three additional genes putatively encoding thioesterase, acyl-CoA dehydrogenase and methyltransferase (Table 1).

**Table 1:** Proposed Functions of the Cholesterol Oxidase Operon (cho) Gene Products.

	Protein	Size (aa)	Function	Closest Sequence Similarity (swissprot)		
				Protein, Origin	Cov/Id (%)	Accession No.
1	ChoR1	204	Transcriptional regulator	ComA, Bacillus subtilis	32/32	P14204
2	ChoD	547	Cholesterol oxidase	ChoA, Streptomyces sp. SA-COO	100/82	P12676
3	ORF A	255	Thioesterase	PikAV, Streptomyces venezuelae	97/52	Q9ZGI1
4	ChoR2	187	Transcriptional regulator	PadR, Bacillus subtilis	96/28	P94443
5	ORF B	574	Acyl-CoA dehydrogenase	Scad, Megasphaera elsdenii	33/27	Q06319
6	ORF C	226	Methyltransferase	BQ2027_MB0092, Mycobacterium bovis	49/40	P65347

### Enzyme kinetics of recombinant ChoD

**Figure 3:**
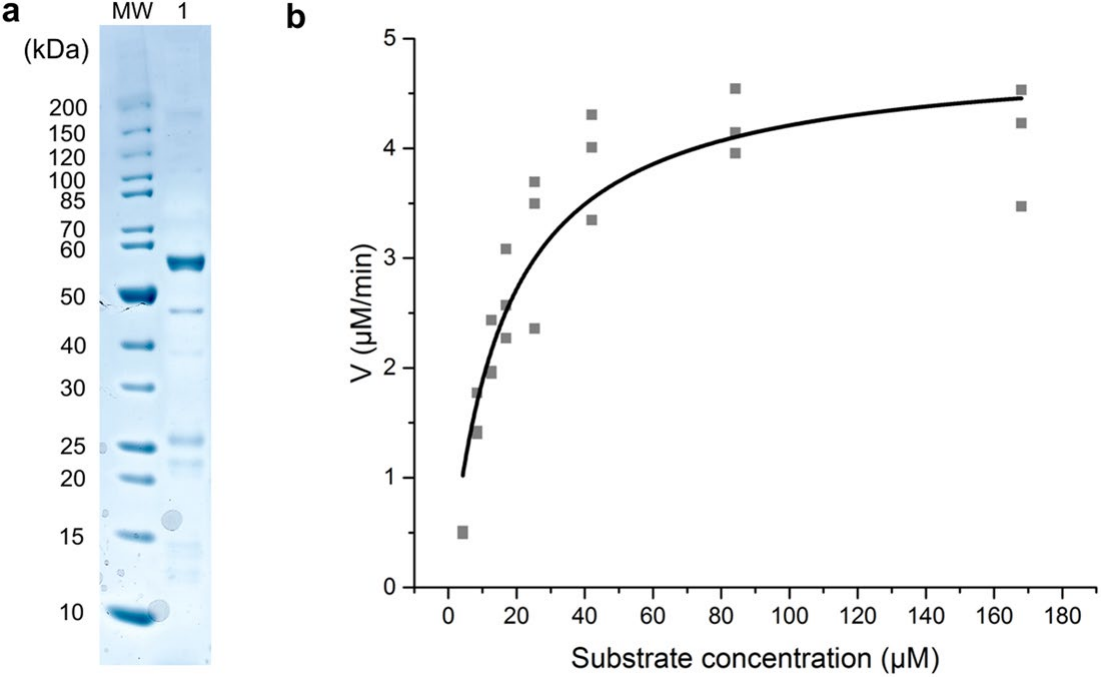
SDS-PAGE analysis of purified ChoD and spectrophotometric determination of enzyme kinetics for ChoD. (**a**) The SDS gel (cropped) was stained with Coomassie Blue and the original gel is presented in Supplementary Fig. S1. Lane MW: protein marker, Lane 1: purified ChoD. (**b**) Spectrophotometric assays were done in triplicate (grey squares) at seven different concentrations of cholesterol.

In order to characterize ChoD, we ordered a synthetic gene codon optimized for expression in *E*. *coli* and cloned it in a modified pBAD vector. The N-terminally histidine-tagged ChoD was produced intracellularly in *E*. *coli*, possibly due to an impaired TAT-transport, and purified to near homogeneity by affinity chromatography (Figs 3a and S1). The enzyme activity was monitored spectrophotometrically by determining H_2_O_2_ concentration, which is formed during non-enzymatic oxidation of reduced flavin in the catalytic cycle, using a colour based reaction with ABTS (2,2′-azino-bis(3-ethylbenzthiazoline-6-sulfonic acid)) at 405 nm (Fig. 1). The progression curves displayed first-order kinetics leading to the determination of basic kinetic parameters for *k*_cat_ (10.35 s^−1^) and *K*_m_ (15.91 µM) (Fig. 3b). The affinity of ChoD towards cholesterol was higher than what has been reported for commercially available cholesterol oxidases from *Brevibacterium* (23 mM), *Streptomyces* (0.2 mM), *Cellulomonas* (84 µM) or *Pseudomonas* (61 µM)^28^. The *K*_m_ of ChoD from *Streptomyces lavendulae* YAKB-15 resided between the values of the two enzymes from *Streptomyces* sp. SA-COO^21^ (3 µM) and *B*. *sterolicum* (>100 µM) for which crystal structures have been determined^29^. Furthermore, the substrate affinity of ChoD from *Streptomyces lavendulae* YAKB-15 is higher than that of the recently reported *Streptomyces* isolate, *S*. *aegyptia* NEAE 102 (152 µM)^30^. However, it should be noted that solubility issues and the use of detergents have been shown to have great influence on kinetic parameters of cholesterol oxidases, which makes comparing the properties of enzymes from various sources challenging^31,32^.

### Heterologous production of ChoD in *S. lividans* TK24 and *S. albus* J1074

In order to improve the production of ChoD, we opted to utilize two widely used and well-characterized *Streptomyces* hosts, *S*. *lividans* TK24^20^ and *S*. *albus* J1074^20^, and two distinct vectors to drive the expression of the ChoD. For expression in *S*. *lividans* TK24, *choD* was cloned in the multi-copy plasmid pIJE486^20^ under the strong constitutive promoter *ermEp* by protoplast transformation. For expression in *S*. *albus* J1074, we elected to use the integrative single copy-number plasmid pS-GK, which is based on the pSET-152^20^ plasmid but contains a strong synthetic promoter SP44^33^, introduced into *Streptomyces* by intergeneric conjugation from *E*. *coli* ET12567/pUZ8002.

**Figure 4:**
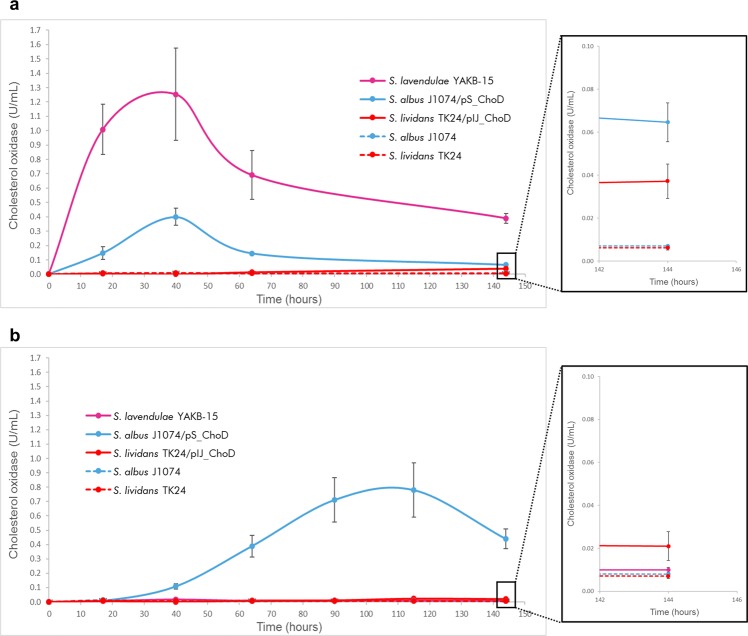
Time course analysis of the production of ChoD by *S*. *lavendulae* YAKB-15 wild type, and the engineered heterologous hosts *S*. *albus* J1074/pS_ChoD and *S*. *lividans* TK24/pIJ_ChoD. Enzyme activity detected from cells grown (**a**) in Y medium with whole yeast and (**b**) in YE medium with yeast extract.

The native strain, *S*. *lavendulae* YAKB-15, produced ChoD rapidly, with the highest activity being 1.25 U/mL at 40 hours in Y medium containing whole yeast cells, whereas no production could be observed in YE medium (Fig. 4), where the whole yeast cells were replaced by yeast extract. *S*. *albus* J1074 produced ChoD in a similar fashion, but with lower activity (0.4 U/mL) in Y medium (Fig. 4a). In YE medium *S*. *albus* J1074 was the only strain able to produce significant amounts of ChoD, with the highest activity at 115 hours and a maximum of 0.78 U/mL (Fig. 4b). Curiously, *S*. *lividans* TK24 produced very little ChoD in both Y and YE medium (Fig. 4).

The production levels of the native strain, *S*. *lavendulae* YAKB-15, and the overexpression strain, *S*. *albus* J1074, are in line with previously reported levels from *Streptomyces* and *Rhodococcus*, which range from 0.2 U/mL to 9.75 U/mL^18,34–40^. However, it should be noted that in many previous reports the medium was extensively optimized to increase production levels. Furthermore, *S*. *lavendulae* YAKB-15 has the highest basal level of activity (1.25 U/mL) for a cell-associated cholesterol oxidase from Actinomycetales (*Rhodococcus*, 0.75 U/mL)^37^.

### Properties of heterologously produced ChoD in *S. albus* J1074

**Figure 5:**
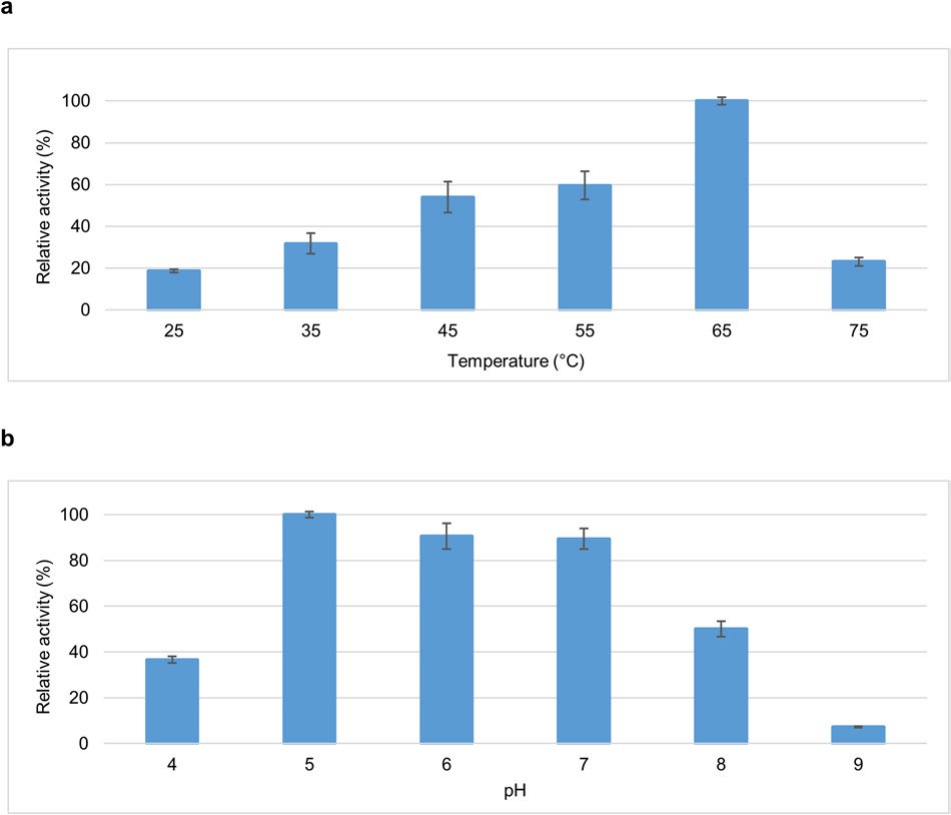
Optimal temperature and pH of ChoD produced in the overexpression host *S*. *albus* J1074/pS_ChoD. Triplicate measurements of (**a**) relative activity based on temperature and (**b**) relative activity based on pH.

The activity of cell-associated ChoD extract produced by the overexpression strain *S*. *albus* J1074/pS_ChoD was characterized using different temperatures and pH. The optimal temperature and pH were determined from a range of 25–75 °C and 4–9, respectively (Fig. 5). The optimal temperature was 65 °C and was dramatically lower (<60%) at any other tested temperature (Fig. 5a). The optimal pH was 5, although pH 6 and pH 7 both had 90% relative activity (Fig. 5b). Both the temperature and pH optima were in line with previously reported cholesterol oxidases as summarized by El-Naggar *et al*.^30^.

### Concluding remarks

In this study we have identified and characterized a cell-associated cholesterol oxidase, ChoD, from *S*. *lavendulae* YAKB-15. To the best of our knowledge ChoD has the highest cholesterol affinity (K_m_ 15.91 µM) and the highest basal activity (1.25 U/mL) of a cell-associated cholesterol oxidase. The optimum temperature and pH was 65 °C and 5, respectively. The presence of the TAT signal indicates that the protein is likely to be produced intracellularly in order to recruit FAD and is exported outside the cell membrane as a fully matured enzyme through the transport system. Since only minor enzymatic activity can be detected from fermentation broths, unlike in the case of other cholesterol oxidase proteins from *Streptomyces*, it is likely that the protein from *S*. *lavendulae* YAKB-15 becomes associated with components of the cell wall. Notably, after heterologous expression of the *choD* in *S*. *lividans* TK24 and *S*. *albus* J1074 the ChoD activity was still associated with the cell fraction and not the supernatant. The fact that *S*. *lavendulae* YAKB-15 only produces ChoD in the presence of whole-yeast raises the possibility that the strain utilizes ChoD as a signalling molecule to detect *Streptomyces*-fungal interactions. Such a role has been proposed for cholesterol oxidases residing in biosynthetic gene clusters responsible for production of several antifungal polyene macrolides^5^. However, no polyene gene clusters are found near *choD* in *S*. *lavendulae* YAKB-15. Future work currently in progress in our laboratory aims to uncover the role of ChoD in the biology of *S*. *lavendulae* YAKB-15.

## Methods

### Genomic DNA isolation and whole genome sequencing

*S*. *lavendulae* YAKB-15 was grown in 250 mL Erlenmeyer flasks containing 30 mL of GYM medium consisting of glucose 4 g/L, yeast extract 4 g/L, malt extract 10 g/L and 0.5% glycine. The pH of the medium was adjusted to 7.2 and the culture was incubated on a rotary shaker (300 rpm) at 30 °C for 96 h. Genomic DNA was extracted using the method from Nikodinovic *et al*. with slight modifications^41^. The DNA was sent to Eurofins Genomics (Ebersberg, Germany) for PCR-free shotgun library preparation (Illumina) and sequenced using MiSeq v3 producing 2 × 300 bp paired-end reads (Illumina).

The quality of the reads was manually checked before and after trimming and error correction using FASTQC (v0.11.2)^42^. The reads were normalized using BBNorm. Then the reads were error corrected and assembled using A5-miseq (v20150522)^43^, contiguated with ABACAS (v1.3.1)^44^ using *Streptomyces albus* NK660 (CP007574.1) as the reference, and the gaps were filled using IMAGE (v2.4.1)^45^. The final assembly was annotated using RAST^46^ and evaluated for completeness using BUSCO (v1.22)^47^. All programs were run with the default parameters on the CSC – IT Center for Science’s Taito super-cluster (Espoo, Finland). The final assembly was deposited in the National Center for Biotechnology Information (NCBI) database under the accession number SMSN00000000.

### *In silico* analysis of cholesterol oxidase from *S. lavendulae* YAKB-15

The *choD* gene was identified in the assembly of *S*. *lavendulae* YAKB-15 using local Protein BLAST^48^ and UniProt sequence P12676 as a query. The sequence was further analysed by comparing important catalytic residues to the found gene^31^. This gene was targeted for cloning and recombinant expression in three different systems.

### Expression systems

Three different expression systems were used to overproduce and quantify the production of ChoD via standard cloning methods^20^. First, an *E*. *coli* codon optimized synthetic *choD* gene was ordered (ThermoFisher Scientific) and cloned in a pBADΔHis^49^ expression plasmid using *Bgl*II/*Hind*III restriction sites, creating pBAD_ChoD, which was transformed into *E*. *coli* TOP10 (Invitrogen). Then the native *choD* gene was PCR amplified using the primer pair 5′-GCGTCTAGAGAAGCTCAGGAGCAACAGCG-3′ (*Xba*I site underlined) and 5′-CGAAGCTTGGATCCTCAGGAACCCGCGATGTCC-3′ (*Hind*III and *Bam*HI sites underlined) from genomic DNA using Phusion high-fidelity DNA polymerase (ThermoFisher Scientific) and cloned in pUC18 (ThermoFisher Scientific) using *Xba*I/*Hind*III restriction sites and then transformed into *E*. *coli* TOP10 (Invitrogen). The DNA sequence of the cloned gene was confirmed by sequencing before subsequent subcloning. Second, the native *choD* gene was digested from the pUC18 cloning plasmid and ligated using the same restriction sites (*Xba*I/*Hind*III) in the expression plasmid pIJE486^50^, creating pIJ_ChoD, and then protoplast transformed into the expression host *S*. *lividans* TK24. Third, the native *choD* gene was digested from the pUC18 expression plasmid using *Xba*I/*Bam*HI restriction sites and ligated in a modified pSET152^51^ expression vector using *Bcu*I/*Bam*HI restriction sites, creating pS_ChoD, and then transformed into *E*. *coli* ET12567/pUZ8002^20^, which was then conjugated into the expression host *S*. *albus* J1074. The plasmid pS_ChoD contained *choD* and superfolder green fluorescence protein (sfGFP) genes with the corresponding ribosomal binding sites under the control of the strong synthetic promoter SP44^33^. To avoid promoter leakage due to the read-through from the upstream genes (i.e. bacteriophage phi31 integrase and apramycin resistance genes) two strong terminator, a synthetic T4 kurz^52^ and a natural terminator ECK120029600^53^ were placed upstream of the promoter.

### Bacterial strains and culture conditions

*S*. *lavendulae* YAKB-15 was obtained from the Russian Collection of Agricultural Microorganisms (RCAM). *S*. *lividans* TK24 and *S*. *albus* J1074 originate from the John Innes Centre^20^. *E*. *coli* TOP10 (Invitrogen) was used for production of the histidine tagged ChoD.

*S*. *lavendulae* YAKB-15 and *S*. *albus* J1074/pS_ChoD were first grown on solid P medium containing 1 g/L peptone, 4.55 g/L glucose anhydrase, 0.4 g/L MgSO_4_ * 7H_2_O, 0.4 g/L K_2_HPO_4_, 22 g/L agar, and 100 g/L potato juice, until they sporulated. *S*. *lividans* TK24/ pIJ_ChoD was grown on ISP4 with 50 μg/mL thiostrepton until it sporulated. Spores were inoculated into 25 mL liquid medium, either Y medium or YE medium, in 250 mL Erlenmeyer flasks; *S*. *lividans* TK24 also contained 50 μg/mL thiostrepton. Y medium contains 9.1 g/L glucose anhydrase, 2 g/L NH_4_NO_3_, 2 g/L CaCO_3_, and 26 g/L common bakery yeast and YE medium is the same except yeast extract substituted common bakery yeast. Liquid pre-cultures were grown at 30 °C shaking at 300 rpm for 24 hours. These pre-cultures were used to inoculate main cultures in triplicates.

For production of ChoD in *E*. *coli* TOP10/pBAD_ChoD, 2 × 500 mL of 2 x TY medium (tryptone 16 g/L, yeast extract 10 g/L, NaCl 5 g/L) or TB medium (tryptone 20 g/L, yeast extract 24 g/L, glycerol 4 mL/L, phosphate buffer (0.17 M KH_2_PO_4_ and 0.72 M K_2_HPO_4_) 100 mL/L) with 100 µg/mL of ampicillin were inoculated with 5 mL of pre-culture per flask. Cultures were cultivated at 37 °C for 3 hours with 250 rpm shaking and were induced with 0.02% L-(+)-Arabinose when the OD_600_ was 1.15. After induction, the cultivation was continued for 15.5 hours at 25 °C with 200 rpm shaking. The cells were harvested by centrifugation at 12,000 × *g* for 30 minutes at 4 °C resulting in wet cell weight of 4.3 g.

For characterization of ChoD properties *S*. *albus* J1074/pS_ChoD was pre-cultured in 15 mL of TSB medium containing 17 g/L tryptone, 3 g/L soy, 5 g/L NaCl, 2.5 g/L K_2_HPO_4_, and 2.5 g/L glucose inoculated from ISP4 spore plates and grown at 30 °C for 24 hours at 300 rpm. The main cultures were also grown in 15 mL of TSB, using 1 mL of pre-culture as inoculum, for at 30 °C for 40 hours at 300 rpm.

### Purification of recombinant proteins

For purification of histidine tagged ChoD, the cells were suspended in 3 mL wash buffer (K_2_HPO_4_ 50 mM, imidazole 5 mM, NaCl 50 mM, 10% glycerol) per gram of cells. The cells were sonicated with a cycle of 11 s of sonication and 40 s of rest on ice. The cycle was repeated 13 times (Sonicator MSE soniprep 150 with max amplitude). 1% of Triton X-100 was added after sonication to the supernatant. Samples were centrifuged 19,000 × *g* at 4 °C for 30 minutes and the supernatant was collected. The supernatant was mixed with 1 mL of TALON affinity resin (GE healthcare) and it was gently shaken for 60 minutes. The resin was washed with 5 mL of wash buffer and the protein was eluted with 2.5 mL of elution buffer (K_2_HPO_4_ 50 mM, imidazole 250 mM, NaCl 50 mM, 10% glycerol). The buffer was changed to storage buffer (K_2_HPO_4_ 50 mM, NaCl 50 mM, 10% glycerol) using a PD-10-column following manufacturer’s instructions. Finally, the glycerol concentration was increased to 40% and purified ChoD was stored at −20 °C. Purified ChoD was evaluated by SDS-PAGE 10%.

### Analysis of cholesterol oxidase activity and enzyme kinetics

ChoD activity was measured spectrophotometrically by the modified method of Sasaki *et al*. The stoichiometric formation of H_2_O_2_ during the oxidation reaction of cholesterol was monitored with ABTS (2,2′-azino-bis(3-ethylbenzthiazoline-6-sulfonic acid)) at 405 nm. To determine the cell-bound ChoD, cultures were centrifuged at 15,000 × g for 10 min. The cell pellet was resuspended in extraction buffer (0.15% Tween 80 in 50 mM phosphate buffer solution) and mixed for 30 minutes at 24 °C. The suspension was centrifuged at 15,000 × *g* and ChoD activity was measured from the supernatant. The activity assay mixture contained 120 μL Triton X-100 (0.05%) in 50 mM sodium-potassium phosphate buffer (pH 7), 10 μL ABTS (9.1 mM in MQ H_2_O), 2.5 μL cholesterol in ethanol (1 mg/mL), 1.5 μL horseradish peroxidase solution (150 U/mL) and 20 μL of the extract preparation in a total volume of 154 μL. The spectrophotometric cholesterol activity assay was carried out in a 96-well plate. One unit of enzyme was defined as the amount of enzyme that forms 1 μmol of H_2_O_2_ per minute at pH 7.0 and 27 °C.

ChoD optimal activity for various pH levels was determined as above with only changes in the buffer as needed for specific pH tests as follows: 50 mM citrate buffer (pH 4–5), 50 mM potassium phosphate buffer (pH 6–7), and 50 mM Tris-HCl buffer (pH 8–9). For optimal temperature activity, temperatures between 25 and 75 °C were obtained using heat blocks. Each condition was tested in triplicates.

For analysis of enzyme kinetics, 8.4 nM ChoD was utilized to probe reaction velocities with eight substrate concentrations ranging between 4–168 µM cholesterol in triplicates. The initial rate of the reaction was calculated from derivatives of progression curves (six initial measurement points over 25 s) and referenced to a H_2_O_2_ standard curve.

## Supplementary information

Supporting information for article

## References

[1] Pollegioni L, Piubelli L, Molla G (2009). Cholesterol oxidase: biotechnological applications. FEBS J.

[2] Doukyu N (2009). Characteristics and biotechnological applications of microbial cholesterol oxidases. Appl. Microbiol. Biotechnol..

[3] Bavari S (2002). Lipid raft microdomains: a gateway for compartmentalized trafficking of ebola and marburg viruses. J. Exp. Med..

[4] Purcell JP (1993). Cholesterol oxidase: a potent insecticidal protein active against boll weevil larvae. Biochem. Biophys. Res. Commun..

[5] Mendes MV (2007). Cholesterol oxidases act as signaling proteins for the biosynthesis of the polyene macrolide pimaricin. Chem. Biol..

[6] Kumari L, Kanwar SS (2012). Cholesterol oxidase and its applications. Adv. Microbiol..

[7] Ahmad S, Goswami P (2014). Application of chitosan beads immobilized *Rhodococcus* sp. NCIM 2891 cholesterol oxidase for cholestenone production. Process Biochem..

[8] Liu J (2014). Cholesterol oxidase from *Bordetella* species promotes irreversible cell apoptosis in lung adenocarcinoma by cholesterol oxidation. Cell Death Dis..

[9] El-Naggar NE-A, Soliman HM, El-Shweihy NM (2018). Extracellular cholesterol oxidase production by *Streptomyces aegyptia*, *in vitro* anticancer activities against rhabdomyosarcoma, breast cancer cell-lines and *in vivo* apoptosis. Sci. Rep.

[10] Lolekha PH, Jantaveesirirat Y (1992). *Streptomyces*: a superior source for cholesterol oxidase used in serum cholesterol assay. J. Clin. Lab. Anal..

[11] Stadtman T, Cherkes A, Anfinsen C (1954). Studies on the microbiological degradation of cholesterol. J. Biol. Chem..

[12] Kumari L, Shamsher KS (2015). Cholesterol oxidase: role in biotransformation of cholesterol. J. Appl. Biol. Biotechnol.

[13] Barnett JP, Eijlander RT, Kuipers OP, Robinson C (2008). A minimal Tat system from a Gram-positive organism. J. Biol. Chem..

[14] Berks BC, Palmer T, Sargent F (2003). The Tat protein translocation pathway and its role in microbial physiology. Adv. Microb. Physiol.

[15] Widdick DA (2006). The twin-arginine translocation pathway is a major route of protein export in *Streptomyces coelicolor*. Proc. Natl. Acad. Sci..

[16] Petrova LI, Podsukhina GM, Dikun TA, Selezneva AA (1979). Conditions for isolation of cholesterol oxidase from Actinomyces lavendulae mycelia. Prikl. Biokhim. Mikrobiol..

[17] Buldakova, T. & Kolodyaznaya, V. Использование альтернативных источников органического азота при биосинтезе холестеролоксидаза Utilization of alternative organic nitrogen sources in cholesterol oxidase biosynthesis. In *VII International Research Conference ‘Actual Scientific Achievements - 2012’* 35–38 (2012).

[18] Varma R, Nene S (2003). Biosynthesis of cholesterol oxidase by *Streptomyces lavendulae* NCIM 2421. Enzyme Microb. Technol..

[19] Ishizaki T, Hirayama N, Shinkawa H, Nimi O, Murooka Y (1989). Nucleotide sequence of the gene for cholesterol oxidase from a *Streptomyces* sp. J. Bacteriol..

[20] Kieser, T., Bibb, M., Buttner, M., Chater, K. & Hopwood, D. *Practical Streptomyces Genetics*. (The John Innes Foundation, 2000).

[21] Yue QK, Kass IJ, Sampson NS, Vrielink A (1999). Crystal structure determination of cholesterol oxidase from *Streptomyces* and structural characterization of key active site mutants. Biochemistry.

[22] Cavener DR (1992). GMC oxidoreductases. A newly defined family of homologous proteins with diverse catalytic activities. J. Mol. Biol..

[23] Wierenga RK, Drenth J, Schulz GE, Huber R (1983). Comparison of the three-dimensional protein and nucleotide structure of the FAD-binding domain of p-hydroxybenzoate hydroxylase with the FAD- as well as NADPH-binding domains of glutathione reductase. J. Mol. Biol..

[24] Chen J, Xie J (2011). Role and regulation of bacterial LuxR-like regulators. J. Cell. Biochem..

[25] Isom CE (2016). Crystal structure and DNA binding activity of a PadR family transcription regulator from hypervirulent *Clostridium difficile* R20291. BMC Microbiol..

[26] Anton N, Mendes MV, Martin JF, Aparicio JF (2004). Identification of PimR as a positive regulator of pimaricin biosynthesis in *Streptomyces natalensis*. J. Bacteriol..

[27] Vicente CM (2014). PAS-LuxR transcriptional control of filipin biosynthesis in *S*. *avermitilis*. Appl. Microbiol. Biotechnol..

[28] Srisawasdi P, Jearanaikoon P, Kroll MH, Lolekha PH (2005). Performance characteristics of cholesterol oxidase for kinetic determination of total cholesterol. J. Clin. Lab. Anal..

[29] Vrielink A, Lloyd LF, Blow DM (1991). Crystal structure of cholesterol oxidase from *Brevibacterium sterolicum* refined at 1.8 Å resolution. J. Mol. Biol..

[30] El-Naggar NEA, Deraz SF, Soliman HM, El-Deeb NM, El-Shweihy NM (2017). Purification, characterization and amino acid content of cholesterol oxidase produced by *Streptomyces aegyptia* NEAE 102. BMC Microbiol..

[31] Vrielink A, Ghisla S (2009). Cholesterol oxidase: Biochemistry and structural features. FEBS J.

[32] Reiss, R., Faccio, G., Thöny-Meyer, L. & Richter, M. Cloning, expression and biochemical characterization of the cholesterol oxidase CgChoA from *Chryseobacterium gleum*. *BMC Biotechnol*. **14** (2014). 10.1186/1472-6750-14-46 PMC4053396 24885249

[33] Bai C (2015). Exploiting a precise design of universal synthetic modular regulatory elements to unlock the microbial natural products in *Streptomyces*. Proc. Natl. Acad. Sci..

[34] Pathak L (2015). Artificial intelligence versus statistical modeling and optimization of cholesterol oxidase production by using *Streptomyces* sp. PLoS One.

[35] Niwas R, Singh V, Singh R, Tripathi D, Tripathi CKM (2013). Production, purification and characterization of cholesterol oxidase from a newly isolated *Streptomyces* sp. World J. Microbiol. Biotechnol..

[36] Fernández de las Heras L (2011). ChoG is the main inducible extracellular cholesterol oxidase of *Rhodococcus* sp. strain CECT3014. Microbiol. Res..

[37] Ahmad S, Goswami P (2013). Enhanced production of cell-bound cholesterol oxidase from *Rhodococcus* sp. NCIM 2891 by the statistical method. Ann. Microbiol..

[38] Chauhan AK, Survase SA, Kishenkumar J, Annapure US (2009). Medium optimization by orthogonal array and response surface methodology for cholesterol oxidase production by *Streptomyces lavendulae* NCIM 2499. J. Gen. Appl. Microbiol.

[39] Moradpour Z, Ghasemian A, Safari A, Mohkam M, Ghasemi Y (2013). Isolation, molecular identification and statistical optimization of culture condition for a new extracellular cholesterol oxidase-producing strain using response surface methodology. Ann. Microbiol..

[40] El-Naggar NE-A, El-Shweihy NM, El-Ewasy SM (2016). Identification and statistical optimization of fermentation conditions for a newly isolated extracellular cholesterol oxidase-producing *Streptomyces cavourensis* strain NEAE-42. BMC Microbiol..

[41] Nikodinovic J, Barrow KD, Chuck JA (2003). High yield preparation of genomic DNA from *Streptomyces*. Biotechniques.

[42] Andrews, S. FASTQC a quality control tool for high throughput sequence data. *Babraham Institute* (2015). Available at, http://www.bioinformatics.babraham.ac.uk/projects/fastqc/Help/3 Analysis Modules/. (Accessed: 9th February 2016).

[43] Coil D, Jospin G, Darling AE (2015). A5-miseq: an updated pipeline to assemble microbial genomes from Illumina MiSeq data. Bioinformatics.

[44] Assefa S, Keane TM, Otto TD, Newbold C, Berriman M (2009). ABACAS: algorithm-based automatic contiguation of assembled sequences. Bioinformatics.

[45] Tsai IJ, Otto TD, Berriman M (2010). Improving draft assemblies by iterative mapping and assembly of short reads to eliminate gaps. Genome Biol..

[46] Brettin T (2015). RASTtk: A modular and extensible implementation of the RAST algorithm for building custom annotation pipelines and annotating batches of genomes. Sci. Rep.

[47] Simão FA, Waterhouse RM, Ioannidis P, Kriventseva EV, Zdobnov EM (2015). BUSCO: assessing genome assembly and annotation completeness with single-copy orthologs. Bioinformatics.

[48] Camacho C (2009). BLAST+: architecture and applications. BMC Bioinformatics.

[49] Kallio P, Sultana A, Niemi J, Mäntsälä P, Schneider G (2006). Crystal structure of the polyketide cyclase AknH with bound substrate and product analogue: implications for catalytic mechanism and product stereoselectivity. J. Mol. Biol..

[50] Ylihonko K, Tuikkanen J, Jussila S, Cong L, Mäntsälä P (1996). A gene cluster involved in nogalamycin biosynthesis from *Streptomyces nogalater*: sequence analysis and complementation of early-block mutations in the anthracycline pathway. MGG Mol. Gen. Genet..

[51] Flett F, Mersinias V, Smith CP (2006). High efficiency intergeneric conjugal transfer of plasmid DNA from Escherichia coli to methyl DNA-restricting streptomycetes. FEMS Microbiol. Lett.

[52] Horbal L, Siegl T, Luzhetskyy A (2018). A set of synthetic versatile genetic control elements for the efficient expression of genes in Actinobacteria. Sci. Rep.

[53] Chen Y-J (2013). Characterization of 582 natural and synthetic terminators and quantification of their design constraints. Nat. Methods.

