# Supplementary material for: Characterization and overproduction of cell-associated cholesterol oxidase ChoD from Streptomyces lavendulae YAKB-15
Source: Sci Rep. 2019 Aug 14;9:11850. doi: 10.1038/s41598-019-48132-1 (PMC6694107; doi:10.1038/s41598-019-48132-1)

**Characterization and overproduction of cell-associated cholesterol oxidase ChoD from *Streptomyces lavendulae* YAKB-15**

Keith Yamada^1^, Arina Koroleva^1,2^, Mitchell Laughlin^1^, Niko Oksanen^1^, Amir Akhgari^1^, Vera Safronova^3^, Elena Yakovleva^2^, Vera Kolodyaznaya^2^, Tatiana Buldakova^2^ and Mikko Metsä-Ketelä^1^.

^1^ University of Turku, Department of Biochemistry, Turku, Finland.

^2^ Saint Petersburg State Chemical Pharmaceutical University, Department of Biotechnology, Saint Petersburg, Russia.

^3^ All-Russian Research Institute for Agricultural Microbiology, Saint Petersburg, Russia.

**Supplementary Figure 1.** Full SDS gel image of ChoD produced from *E. coli* TOP10/pBAD_ChoD. Lanes 1-5: elution fractions after affinity chromatography of ChoD produced in TB medium. Lane 6-9: fractions of ChoD produced in 2xTY medium. Lane 10-12: supernatant, cell lysate and flow-through, respectively, from production in TB medium. Lane 13-14: supernatant and cell lysate, respectively, from production in 2xTY medium.


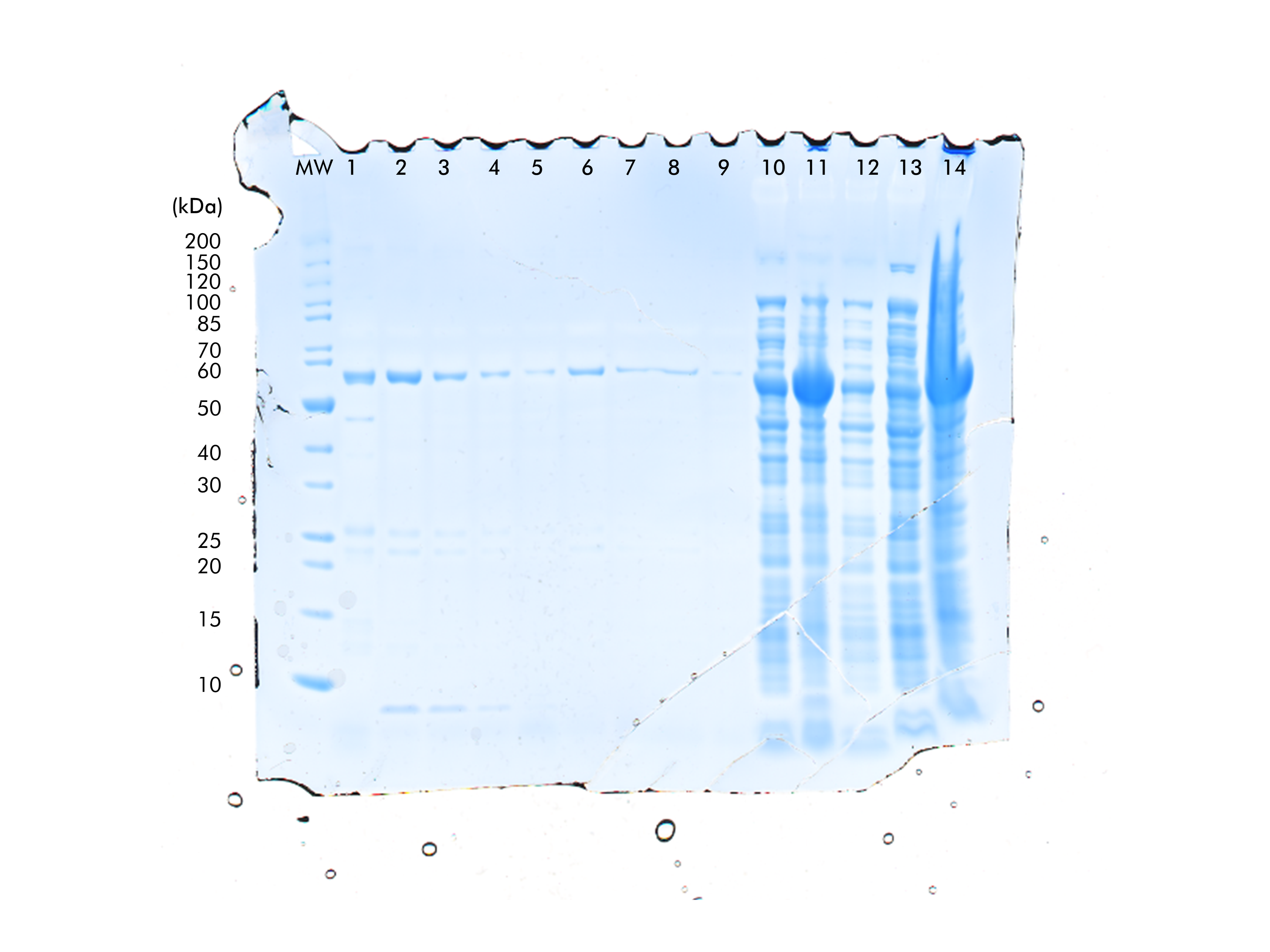

Supplement: Supplementary file 1 — Supporting information for article [file 41598_2019_48132_MOESM1_ESM.docx]
